# Resilience of soybean cultivars to drought stress during flowering and early-seed setting stages

**DOI:** 10.1038/s41598-023-28354-0

**Published:** 2023-01-23

**Authors:** Sadikshya Poudel, Ranadheer Reddy Vennam, Amrit Shrestha, K. Raja Reddy, Nuwan K. Wijewardane, Krishna N. Reddy, Raju Bheemanahalli

**Affiliations:** 1 Department of Plant and Soil Sciences, Mississippi State University, Mississippi State, MS USA; 2 Department of Agricultural & Biological Engineering, Mississippi State University, Mississippi State, MS USA; 3 Crop Production Systems Research Unit, USDA-ARS, Stoneville, MS USA

**Keywords:** Abiotic, Drought, Plant sciences, Plant stress responses

## Abstract

Drought stress during the reproductive stage and declining soybean yield potential raise concerns about yield loss and economic return. In this study, ten cultivars were characterized for 20 traits to identify reproductive stage (R1–R6) drought-tolerant soybean. Drought stress resulted in a marked reduction (17%) in pollen germination. The reduced stomatal conductance coupled with high canopy temperature resulted in reduced seed number (45%) and seed weight (35%). Drought stress followed by rehydration increased the hundred seed weight at the compensation of seed number. Further, soybean oil decreased, protein increased, and cultivars responded differently under drought compared to control. In general, cultivars with high tolerance scores for yield displayed lower tolerance scores for quality content and vice versa. Among ten cultivars, LS5009XS and G4620RX showed maximum stress tolerance scores for seed number and seed weight. The observed variability in leaf reflectance properties and their relationship with physiological or yield components suggested that leaf-level sensing information can be used for differentiating drought-sensitive soybean cultivars from tolerant ones. The study led to the identification of drought-resilient cultivars/promising traits which can be exploited in breeding to develop multi-stress tolerant cultivars.

## Introduction

With the world's population estimated to reach 9.8 billion by 2050, the demand for food crops is increasing^1^. Soybean (*Glycine max* [L.] Merr.) is a leading oilseed crop grown in a wider range of climatic conditions^2,3^. After corn, soybean is the most widely planted crop in the US, accounting for 32% of the total cultivated land^4^. The ongoing climate changes, characterized by erratic rainfall events, pose a severe threat to food security and have raised concern about their interactive impact on the food chain^5^. Drought stress has been one of the critical factors affecting the stability and productivity of soybean in many regions of the world, including the US^6^. Rainfed agriculture accounts for 90 percent of the US soybean output, accounting for over one-third of the global soybean market^7^. Due to global climate change, erratic rainfall patterns have persistently posed a significant threat to soybean production, especially in rainfed areas^8–10^.

Drought is complex stress that affects various morpho-physiological traits at all growth stages, resulting in considerable economic losses^11^. The water requirements of soybeans double during the reproductive stage compared to the vegetative stage^12^. The frequency and intensity of drought stress are predicted to increase around the highly susceptible growth stages such as flowering and post-flowering in soybean^13,14^. Drought stress at these sensitive stages (R1–R6) significantly reduces yield by affecting the pollen fertility^15,16^, sink size and yield components (seed number per pod and branch)^17,18^. To characterize differences in drought tolerance, many studies have focused on finer scale physiological parameters and reported the decline in stomatal conductance, photosynthesis, and quantum efficiency in field crops^19–21^. Although the soybean response to drought stress may vary with the cultivars, it has been reported that drought stress can induce up to a 40% reduction in yield^22^. Alternatively, previous studies reported the genetic variability in yield determining physiology and growth traits under drought stress ^23–25^. Besides the yield of soybean, seed quality (protein and oil) is an important indicator of high market value which is determined by cultivar and growing conditions. Drought stress not only affects the source development but can cause significant damage to the sink potential by intervening with carbon to nitrogen ratio^26^. For instance, the interaction of drought stress with key metabolic processes negatively influences the quality composition^27^. Generally, reduced nitrogen fixation and partitioning significantly affect metabolism and biosynthesis of protein under drought stress^27–29^. In addition, drought stress triggers dysfunction of the endoplasmic reticulum, which activates the accumulation of misfolded or unfolded protein^30,31^. Although some studies reported variations in seed quality traits under different cultivation systems and environment, the responses of advanced soybean cultivars to drought stress at flowering-early seed setting (R1–R6) remain largely unexplored.

In the context of non-destructive phenotyping, leaf reflectance properties either multispectral or hyperspectral reflectance has been used to monitor changes in crop response to stresses^32–34^. To overcome the challenges associated with traditional phenotyping, vegetation indices (VIs) associated with leaf pigments and physiological parameters are commonly used to study crop responses to stresses ^35,36^. For example, the stressed plant shows higher reflectance in red and lower near-infrared reflectance compared to the non-stressed plant^37^. Different VIs were found to be related to the same physiological trait for a different crop. For instance, the red edge chlorophyll index was strongly associated with leaf chlorophyll in a maize^38^. The normalized pigment chlorophyll ratio index was found to be the best index for measuring peas leaf chlorophyll^39^. Despite reports on soybean responses to drought stress at various growth stages, the application of remote sensing tools to study stress-induced leaf properties has been relatively limited.

In southern states of the US, the flowering and early seed setting stages often exposed to low and erratic rainfall, resulting in yield and quality losses^40^. Therefore, using classical and advanced phenotyping assays, the present study investigated the drought stress induced phenotypic responses of soybean cultivars during flowering-early seed setting stages. The specific objectives of this study were to (i) determine genetic variability in pollen germination and physiological responses of soybean to drought stress, (ii) select key plant health-related vegetation indices, (iii) quantify the impact of drought stress on yield and seed quality, and (iv) identify soybean cultivar (s) inducing greater drought stress resilience.

## Results

### Soybean pollen germination

**Figure 1:**
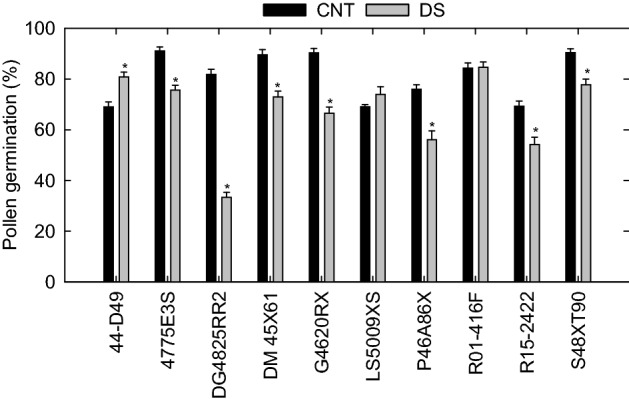
Pollen germination (%) of ten soybean cultivars under control (CNT) and drought stress (DS). The vertical bars represent mean of ten replicates ± SE. *indicates a significant difference between treatments at *p* < 0.05.

Pollen germination was significantly affected by cultivar (*p* ≤ 0.001), treatment (*p* ≤ 0.001), and cultivar × treatment interaction (*p* ≤ 0.001; Table 1). All ten soybean cultivars responded differentially to drought stress (Fig. 1). Under control, pollen germination ranged from 69% (44D49) to 91% (4775E3S), with a mean of 81% (Table 1). Six of the ten cultivars had more than 80% pollen germination under control, whereas only two cultivars recorded pollen germination above 80% under drought stress. Among ten cultivars, DG4825RR2 recorded the highest reduction in pollen germination (59%) under drought stress compared to control, followed by G4620RX (26%). However, in both treatments, cultivars R01-416F and LS5009XS showed no significant difference in pollen germination (Fig. 1). On average, in vitro pollen germination across cultivars was reduced by 17% under drought stress compared to the control.

**Table 1:** Analysis of variance and mean values of physiology, leaf reflectance properties, yield, and seed quality parameters of ten soybean cultivars (C) under control (CNT) and drought stress (DS) treatments (T).

Trait	T	C	T x C	Mean		Correlation
				CNT	DS	CNT vs. DS
Pollen germination (PG, %)	***	***	***	81.14^a^	67.65^b^	0.17^ ns^
Chlorophyll content (Chl, µg cm^−2^)	***	ns	*	28.18^a^	25.67^b^	0.30^ ns^
Flavonoid index	ns	ns	ns	1.02^a^	0.99^a^	0.26^ ns^
Anthocyanin index	ns	ns	ns	0.15^a^	0.15^a^	0.23^ ns^
Nitrogen Balance Index (NBI)	ns	ns	ns	27.82^a^	26.58^a^	0.44^ ns^
Stomatal conductance (gsw, mol m^−2^ s^−1^)	***	***	**	1.02^a^	0.05^b^	0.33 ns
Transpiration (E, mmol m^−2^ s^−1^)	***	*	ns	8.38^a^	1.47^b^	0.40^ ns^
Photosystem II efficiency (PhiPS2)	***	*	ns	0.69^a^	0.6^b^	0.08^ ns^
Canopy temperature (CT, ºC)	***	**	ns	33.7^b^	35.6^a^	0.67*
Specific leaf area (SLA, cm^2^ g^−1^)	***	**	ns	315.83^a^	239.72^b^	0.63*
Normalized Difference Red Edge (NDRE)	ns	**	ns	0.31^a^	0.32^a^	0.20^ ns^
Photochemical Reflectance Index (PRI)	**	ns	ns	0.07^a^	0.06^b^	0.34^ ns^
Transformed Chlorophyll Absorption in Reflectance Index (TCARI)	***	**	ns	0.21^a^	0.18^b^	0.42^ ns^
Visible Atmospherically Resistant Index (VARI)	***	**	ns	0.6^a^	0.50^b^	0.27^ ns^
Plant height (PHT, cm)	***	***	**	92^a^	69^b^	0.90***
Seed number (SN, plant^−1^)	***	***	*	350.74^a^	191.86^b^	0.57^ ns^
Seed weight (SWt., g plant^−1^)	***	***	**	48.18^a^	31.45^b^	0.24^ ns^
Hundred seed weight (HSWt., g)	***	***	***	16.68^a^	13.83^b^	0.69*
Protein (%)	***	***	***	40.11^a^	38.6^b^	0.10^ ns^
Oil (%)	***	***	**	21.87^a^	21.39^b^	0.60*

*, **, and ***, indicate significance levels at *p* < 0.05, *p* < 0.01, *p* < 0.001, respectively. ns, indicates nonsignificance. The mean values were separated using the least significant difference (LSD) test at *p ≤* 0.05. Different letters in superscript indicate the significant treatment effect. The correlation value determines the interdependence between the control and drought stress for the studied parameters.

### Cultivar differences in physiological traits under drought stress

**Figure 2:**
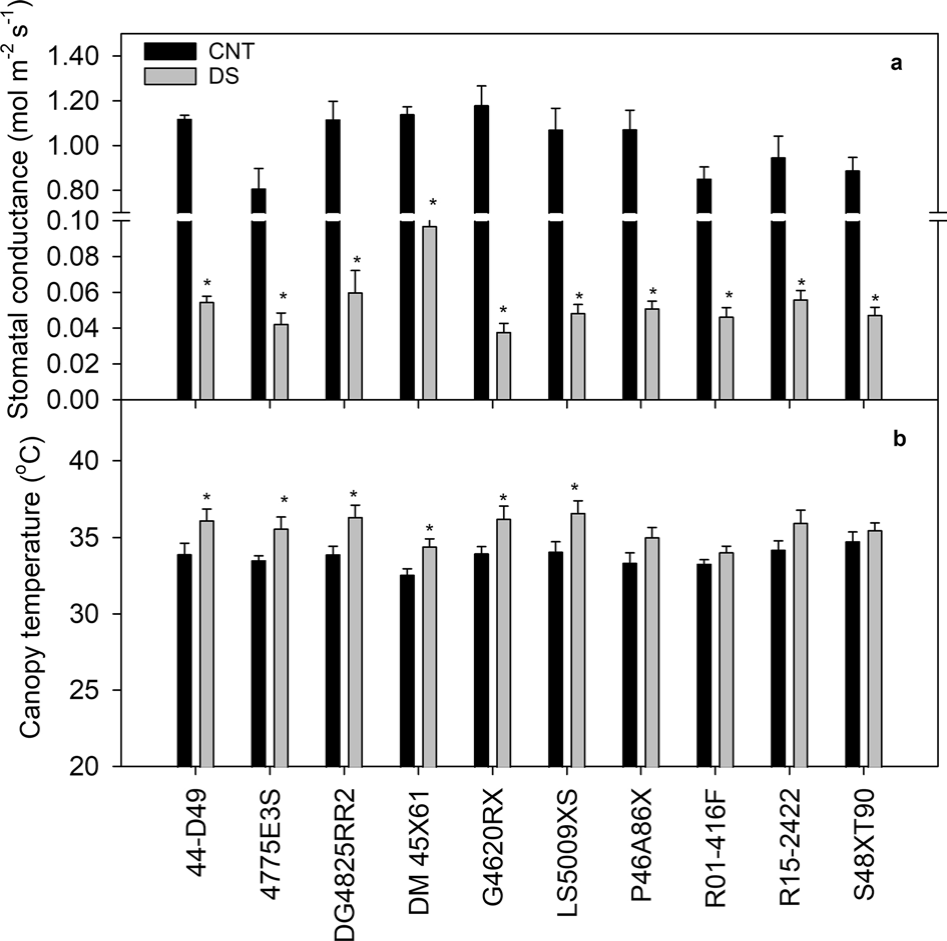
Stomatal conductance (**a**) and canopy temperature (**b**) responses of ten soybean cultivars under control (CNT) and drought stress (DS). The vertical bars represent mean of ten replicates ± SE. *indicates a significant difference between the CNT and DS for the given cultivar at *p* < 0.05.

A significant cultivar (*p* < 0.001), treatment (*p* < 0.001), and cultivar × treatment interaction (*p* < 0.01) effect was recorded for stomatal conductance (Table 1). The stomatal conductance ranged from 0.81 mol m^−2^ s^−1^ for 4775E3S to 1.18 mol m^−2^ s^−1^ for G4620RX under control. Under drought stress, the cultivar G4620RX had the highest decrease in stomatal conductance (97%; Fig. 2a). A significant cultivar (*p* < 0.01) and treatment (*p* < 0.001) effect was recorded for canopy temperature (Table 1). Drought stress increased the midday canopy temperature by 2 °C. Four cultivars showed no significant rise in canopy temperature under drought. Drought-stressed R01-416F plants maintained the lower midday canopy temperature (2.5 °C) compared to DG4825RR2 (Fig. 2b).

**Figure 3:**
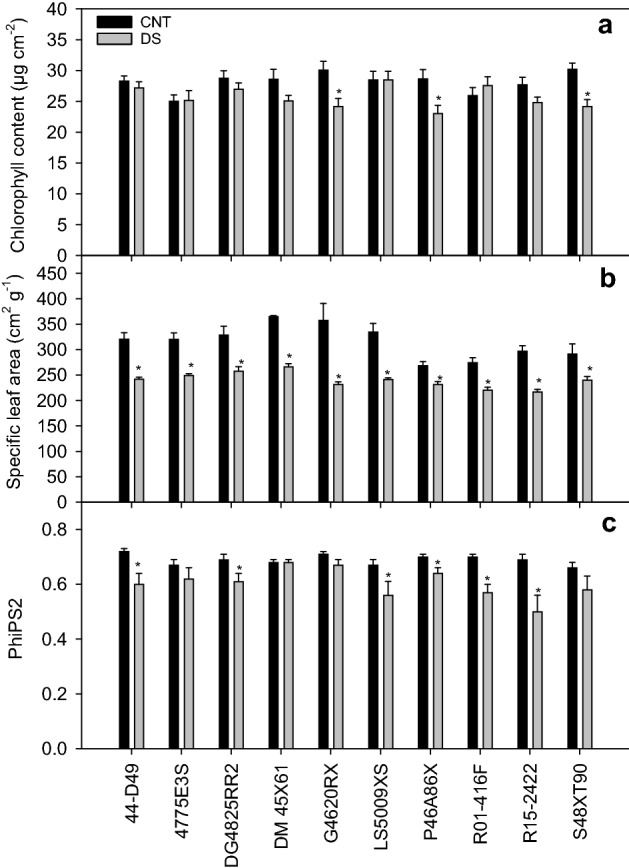
Effect of drought stress on chlorophyll content (**a**), specific leaf area (**b**) and photosystem II efficiency (PhiPS2, **c**) during flowering-early seed setting stage. The vertical bars represent mean of ten replicates ± SE. *indicates a significant difference between the control (CNT) and drought stress (DS) for the given cultivar at *p* < 0.05.

The leaf pigments did not vary significantly across the treatment, cultivar, and treatment × cultivar interaction except for the chlorophyll content (Table  1). Chlorophyll content decreased (~ 9%) under drought stress over control. Among the ten cultivars, S48XT90, G4620RX, and P46A86X showed significant decrease in chlorophyll content under drought stress compared to control (Fig. 3a). A significant cultivar (*p* < 0.01) and treatment (*p* < 0.001) effect was recorded for specific leaf area (Table 1). The result indicated that all the cultivars had a smaller specific leaf area (24% decrease) under drought than the control (Fig. 3b), with the highest reduction in specific leaf area was noted in cultivar G4620RX. In this study, quantum efficiencies of photosynthetic electron transport through photosystem II (PhiPS2) varied significantly across the cultivars (*p* < 0.05) and treatments (*p* < 0.001; Table 1). Under drought stress, PhiPS2 was reduced by 12% compared to the control (Fig. 3c). The cultivars R15-2422 and R01-416F had the highest percentage reduction in PhiPS2 under drought stress. Based on physiological parameters, DM45X61 and 44-D49 displayed better resilience to drought stress than other cultivars.

### Leaf reflectance properties

**Figure 4:**
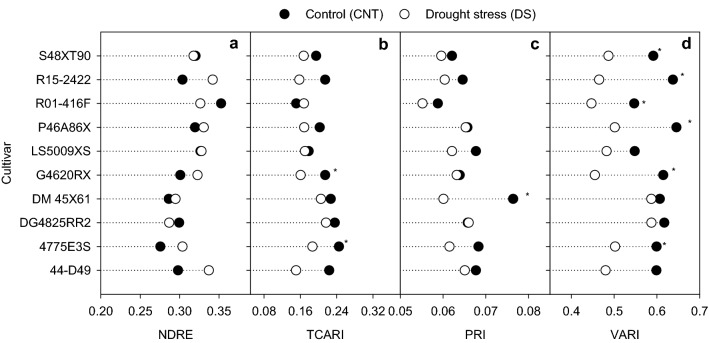
Normalized difference red edge index (NDRE, **a**) transformed chlorophyll absorption in reflectance index (TCARI, **b**), photochemical reflectance index (PRI, **c**), and visible atmospherically resistant index (VARI, d) under control (CNT) and drought stress (DS). *indicates significant difference between treatments for the given cultivar at *p* < 0.05.

To determine the effect of drought stress on plant health, VIs related to pigments and photosynthetic efficiency were used in the study (Table 1). Transformed Chlorophyll Absorption in Reflectance Index (TCARI) and Visible Atmospherically Resistant Index (VARI) were significantly affected by treatment and cultivars (*p* < 0.05 to *p* < 0.001). In contrast, a significant cultivar effect (*p* < 0.01) was observed for Normalized Difference Rededge (NDRE), and significant treatment effect (*p* < 0.01) was observed for Photochemical Reflectance Index (PRI) (Table 1). Under drought stress, VARI was reduced by 17% (*p* < 0.001), followed by TCARI by 16% (*p* < 0.001), and PRI by 7% (*p* < 0.01), while the NDRE was reduced by less than 5% compared to control (Fig. 4). The cultivar DM45X61 which had no change in PhiPS2 between treatments recorded the maximum VARI under drought stress. VARI and PRI were the most effective VIs in detecting differences between treatments and cultivars, suggesting that these VIs could be used for large scale phenotyping.

### Yield components

**Figure 5:**
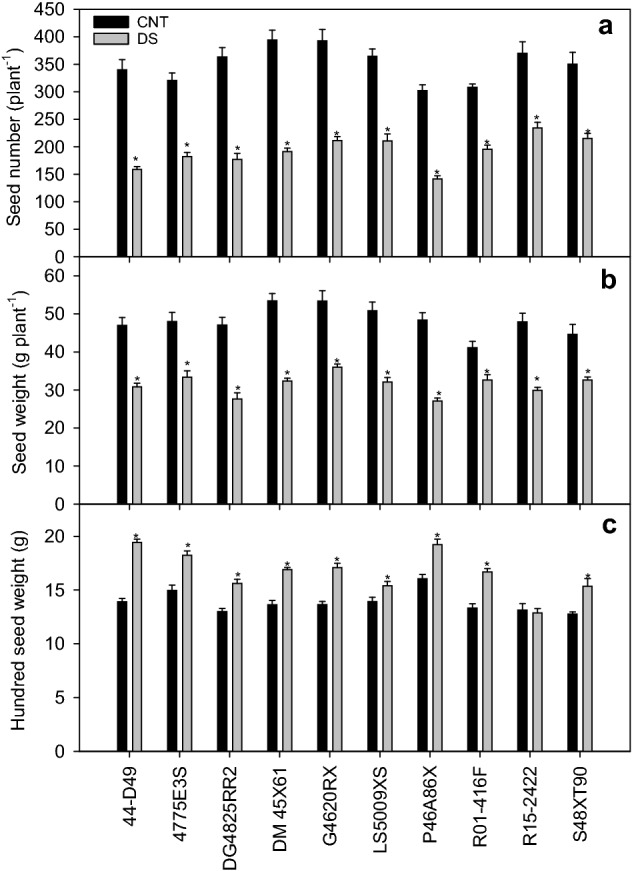
Seed number (plant^−1^, **a**) seed weight (g plant^−1^, **b**), and hundred seed weight (**g**, **c**) of ten soybean cultivars under control (CNT) and drought stress (DS). The vertical bars represent mean of 10 replicates ± SE. *indicates a significant difference between the CNT and DS for the given cultivar at *p* < 0.05.

The yield-related parameters were significantly affected by cultivar, treatment, and cultivar × treatment interaction (Table 1). When averaged across cultivars, drought stress significantly decreased seed number (46%) and weight (35%) compared to the control. A decrease in seed number was relatively less in R15-2422 (34.6%) compared to 44-D49 (52.1%) or P46A86X (52.6%) under drought (Fig. 5a). Likewise, under control, cultivars DM45X61, G4620RX, and LS5009XS recorded the highest seed weight, while under drought stress, cultivars G4620RX, 4775E3S, and S48XT90 were the high yielders (Fig. 5b). Hundred seed weight varied significantly among cultivars (*p* < 0.001), treatments (*p* < 0.001), and cultivar × treatment interaction (*p* < 0.001; Table 1). On average, most cultivars exhibited a higher hundred seed weight under drought stress. Among the cultivars, 44-D49 recorded the highest increase (40%) in hundred seed weight under drought stress compared to control followed by R01-416F (26%) and G4620RX (25%; Fig. 5c). The cultivar R15-2422 with the highest seed number had small-sized seeds. Whereas the cultivar P46A86X with the lowest seed number displayed the highest hundred seed weight/seed size. With the exception of hundred seed weight, yield parameters were significantly reduced under drought stress.

### Seed quality compositions

The seed protein and oil content had a significant variation for treatments *(p* < 0.001), cultivars *(p* < 0.001), and cultivar × treatment interaction *(p* < 0.01; Table 1). The seed protein ranged from 37% for P46A86X to 41% for R15-2422 under control, while under drought stress it ranged from 39% (DG4825RR2) to 42% (4775E3S) (Table 2). Under drought stress, the highest increase in protein content was for cultivar P46A86X (10%) followed by S48XT90 (7%). The seed oil content ranged from 21% for G4620RX to 23% for P46A86X under control (Table 2). On average, the oil content decreased by 2% under drought stress compared to the control. The decrease in oil content was maximum for 44D49 (7%) followed by P46A86X (5%) and S48XT90 (5%) under drought stress.

**Table 2:** Variation in seed protein and oil content  of ten soybean cultivars grown under control and drought stress.

Cultivars	Protein (%)			Oil (%)		
	CNT	DS	Change	CNT	DS	Change
44-D49	38.9 ± 0.3^b^	41.3 ± 0.2^a^	6.3	21.6 ± 0.2^a^	20.2 ± 0.2^b^	− 6.6
4775E3S	38.8 ± 0.5^b^	41.6 ± 0.3^a^	7.3	21.6 ± 0.4^a^	20.8 ± 0.3^a^	− 3.4
DG4825RR2	37.4 ± 0.2^b^	38.4 ± 0.4^a^	2.5	22.2 ± 0.2^a^	22.3 ± 0.2^a^	0.9
DM 45X61	38.4 ± 0.2^b^	39.5 ± 0.2^a^	2.9	22.2 ± 0.3^a^	21.6 ± 0.2^a^	− 2.3
G4620RX	38.4 ± 0.2^b^	40.7 ± 0.3^a^	6.0	21.2 ± 0.3^a^	20.7 ± 0.3^a^	− 2.2
LS5009XS	39.3 ± 0.4^a^	39.6 ± 0.4^a^	1.0	22.1 ± 0.3^a^	22.2 ± 0.3^a^	0.5
P46A86X	36.9 ± 0.2^b^	40.4 ± 0.4^a^	9.5	22.7 ± 0.2^a^	21.5 ± 0.4^b^	− 5.2
R01-416F	39.7 ± 0.4^a^	39.7 ± 0.6^a^	0.1	20.7 ± 0.3^a^	20.8 ± 0.3^a^	0.6
R15-2422	40.6 ± 0.6^a^	39.6 ± 0.4^b^	− 2.3	22 ± 0.3^a^	22.3 ± 0.3^a^	1.6
S48XT90	37.6 ± 0.5^b^	40.3 ± 0.5^a^	7.3	22.5 ± 0.4^a^	21.5 ± 0.2^b^	− 4.5

Values represent mean (*n* = 10) ± SE for the quality parameters of the ten soybean cultivars under control (CNT) and drought stress (DS). Different letters in superscript indicate the significant treatment effect for a given parameter between treatments. Negative value represents a percentage reduction and positive value represents a percentage increase under drought stress compared to control.

### Correlation between traits under control and drought stress

**Figure 6:**
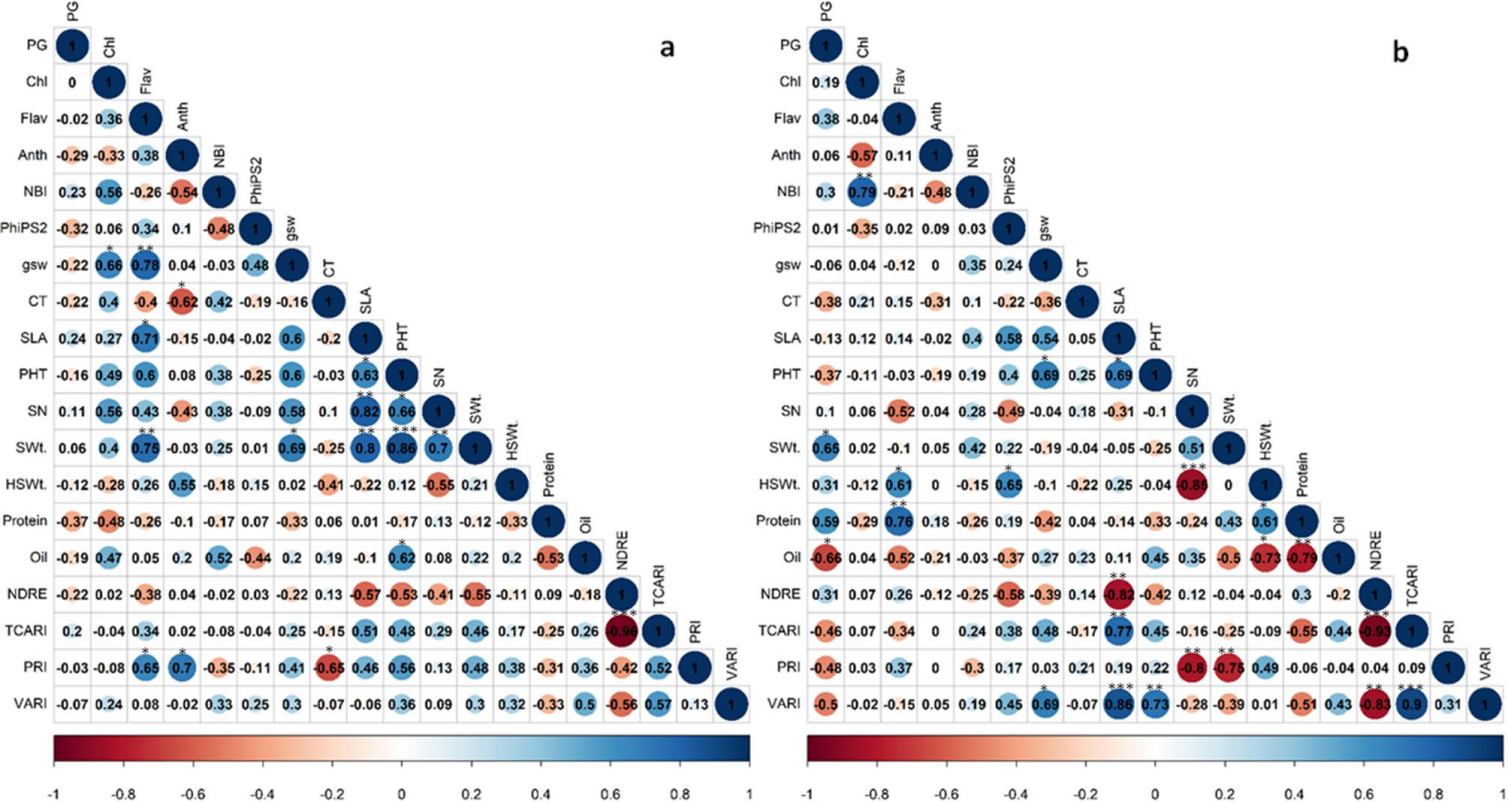
Pearson’s correlation coefficient between pigments, physiology, leaf reflectance properties, yield, and quality parameters under control **﻿(a)** and drought stress **(b)**. The high and low intensity of color represents strong and weak relationships (blue for positive and red for negative) between the two variables, respectively. Values closer to one indicate a strong correlation, and a value closer to zero indicates a weaker relationship between the two variables. *p < 0.05, **p < 0.01, ***p < 0.001 indicate significant correlation between pair of parameters. Traits acronyms are given in Table 1.

Weak relationships between treatments (control vs. drought) for seed number and weight highlight differential responses of soybean cultivars to drought stress. Some traits (canopy temperature, specific leaf area, plant height, hundred seed weight, and oil) showed consistency in performance between treatments (Table 1) but had no correlation with yield under drought. Drought-stressed plants showed a positive correlation between seed number and seed weight (*r* = 0.51), but a weaker relationship than control (Fig. 6). Whereas hundred seed weight showed a strong and negative correlation with seed number under drought stress (*r* =  − 0.85, *p* < 0.001) (Fig. 6b). The drought-stressed plant showed a significant positive relationship between pollen germination and seed weight (*r* = 0.65, *p* < 0.05), such correlation was not observed under control (Fig. 6a). The vegetation index such as PRI was negatively correlated with seed number (*r* =  − 0.8, *p* < 0.01), and seed weight (*r* =  − 0.75, *p* < 0.01), under drought stress (Fig. 6b). Under drought stress, there was a significant negative relationship between hundred seed weight and oil (*r* =  − 0.73, *p* < 0.01), oil and protein content (*r* =  − 0.79, *p* < 0.01). Chlorophyll content and nitrogen balance index (*r* = 0.79, *p* < 0.01) were positively correlated under drought. A positive correlation was observed between stomatal conductance and plant height (*r* = 0.69, *p* < 0.05) under drought stress (Fig. 6b). NDRE showed a significant negative correlation with specific leaf area (*r* =  − 0.82, *p* < 0.01) under drought. Further, VARI showed a significant positive correlation with stomatal conductance (*r* = 0.69, *p* < 0.05), specific leaf area (*r* = 0.86, *p* < 0.001), and plant height (*r* = 0.73; *p* < 0.01) under drought stress.

Under control, stomatal conductance was positively correlated with specific leaf area (*r* = 0.60), plant height (*r* = 0.6), seed number (*r* = 0.58), and seed weight (*r* = 0.69, *p* < 0.05) (Fig. 6a). Seed weight was positively correlated with the flavonoid index (*r* = 0.75, *p* < 0.01), specific leaf area (*r* = 0.8, *p* < 0.01), and seed number (*r* = 0.7, *p* < 0.05) under control. Plant height under control was significantly and positively correlated with seed weight (*r* = 0.86, *p* < 0.001) and seed number (*r* = 0.66, *p* < 0.05). However, under drought stress, this relation was non significant (Fig. 6b). Under control, PRI showed significant correlations with flavonoid index (*r* = 0.65; *p* < 0.05), anthocyanin index (*r* = 0.70; *p* < 0.05) and canopy temperature (*r* =  − 0.65; *p* < 0.05).

### Drought stress tolerance of soybean

**Figure 7:**
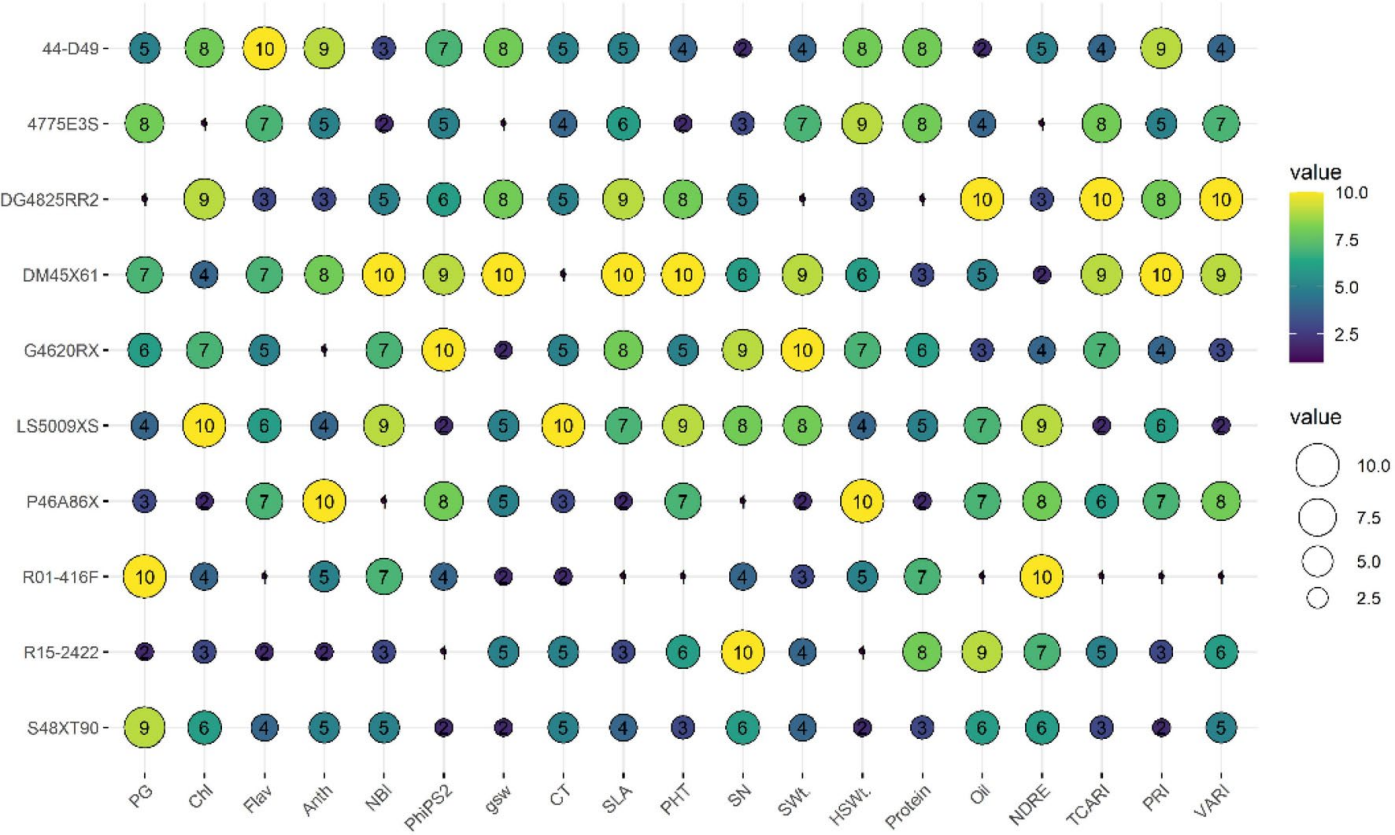
Bubble plot showing the variability in drought stress tolerance ranking of ten soybean cultivars. The high (10) and low (1) rank represents drought tolerant and sensitive cultivar or trait, respectively. Traits acronyms are given in the Table 1.

 Using the stress tolerance index, cultivars were ranked as drought-tolerant (rank 10) or sensitive (rank 1). Two cultivars (LS5009XS, and G4620RX) with maximum tolerance score for seed number recorded maximum seed weight. The cultivar R15-2422 with the highest seed number showed the lowest score for hundred seed weight (Fig. 7). In contrast, the cultivar P46A86X that ranked the lowest tolerance for the seed number displayed a high stress tolerance for the hundred seed weight. The cultivar G4620RX showed a better stress tolerance score for seed number, seed weight, and hundred seed weight. In general, cultivars with high tolerance scores for protein displayed lower tolerance scores for oil content and vice versa except R15-2422 and LS5009XS. DG484RR2 scored the highest rank in oil and the lowest in protein. When protein and oil ranks were combined, R15-2422 had better seed quality with marginal seed weight compared to other cultivars. Based on the average stress tolerance ranks across traits (seed number, seed weight, hundred seed weight, protein, and oil), G4620RX, LS5009XS, and R15-2422 were classified as tolerant.

## Discussion

Some of the changes that drought-stressed plants have undergone during the vegetative stage can be restored once the stress is relieved^41^. Unlike in the vegetative stage, damages caused by stressors at flowering-early seed setting are irreversible and reported to have a higher detrimental effect on economic return. Therefore, the characterization and selection or development of soybean cultivars that can withstand drought stress during the sensitive growth stages is vital for sustaining productivity and ensuring food security. Characterization of soybean cultivars recommended for the southern US climate showed  substantial genetic variability for functional and yield traits under drought stress (Table 1), which can further be utilized to understand the tradeoff between tolerance and yield potential.

In scenarios where soil moisture is insufficient to meet evapotranspiration demands, stomatal conductance decreases and increases the canopy temperature^42–44^. Cultivars with higher stomatal conductance coupled with efficient photosystem II function are important traits that represent drought tolerance in rainfed grown or upland crops^13,45^. Among the ten soybean cultivars, DM45X61 showed such traits under drought, recording high stomatal conductance and nitrogen balance index with relatively low midday canopy temperature (Fig. 7). Furthermore, drought stress-induced changes in leaf reflectance properties (PRI, TCARI, and VARI) supported the manually measured observations (PhiPS2 and canopy temperature), (Fig. 4). However, changes were variable among cultivars with DM45X61 exhibiting better physiology and plant health under drought (Fig. 7). NDRE and pigment measurements were not influenced by treatment, which indicates that a decrease in specific leaf area could lead to a higher concentration of chlorophyll per unit area under drought. Interestingly, cultivars that had high specific leaf area positively correlated with TCRI (*r* = 0.70, *p* < 0.05) or PRI (*r* = 0.71, *p* < 0.05) and negatively with NDRE (*r* =  − 0.78, *p* < 0.05). In addition, VARI was the only index associated with stomatal conductance under drought (Fig. 6b). Unlike other indices, VARI is solely based on the combination of the blue, green, and red bands from the visible region of PAR and was found to be related to moisture content^46^, chlorophyll^47^, and vegetation fraction^48^. Also, it is less sensitive to atmospheric effects. In our study, variability in leaf reflectance properties and their relationship with physiological traits or yield suggested that leaf-level VIs information can be used for differentiating drought-sensitive cultivars from tolerant in soybean.

As soybean plant uses a high volume of water during peak flowering and seed setting, prolonged drought stress adversely impacts its health and productivity^49^. Stressors at the reproductive stage negatively impact reproductive processes such as pollen germination, flower number, and seed production in field crops, including soybean^50,51^. A decline in pollen viability and seed set has been reported under drought stress in maize^44^, rice^52^, and wheat^53^. In our study, pollen germination decreased under drought except in 44-D49 (Fig. 1). A possible explanation for 44-D49's increased pollen germination might be the increased cooling mechanisms in flowers or differential cooling mechanisms through flowers compared to leaves^54^. However, more research is required in this area. Previous study in soybean also reported a decline in flower load, and seed-set percentage when exposed to drought stress during the gametogenesis-bloom period^55,56^. Apparently, the injury to flowers or pollen caused by drought directly affected seed number, particularly in cultivars that are sensitive to drought stress. Although the cultivar R01-416F was superior to other cultivars in pollen germination, the physiological performance was below average under drought-stress conditions (Fig. 7). This resulted in poor yield, making it less tolerant under drought stress among the ten cultivars. It should be noted, however, the relationships between pollen germination and yield components were weaker, suggesting the possibility of improving reproductive tolerance under drought.

It was reported that drought stress during the reproductive stage significantly impacts soybean yield (74% decline) compared to the vegetative phase (28% decline)^41^. On average, reduced stomatal conductance and transpiration coupled with high midday canopy temperature resulted in decline of seed number and weight. A similar result was reported in wheat, where grain yield was reduced by 69% under drought stress^57^. The drought stress-induced reduction in seed number per plant was positively correlated with reductions in seed weight (*r* = 0.67) and hundred seed weight (*r* =  − 0.52). Reduced seed weight did not correlate with hundred seed weight, suggesting that drought followed by rehydration increased seed size at the expense of seed number (Fig. 5c). These findings indicate that soybean plants diverted resources to maintain the growth of pods/seeds that are already formed by reducing the formation of new flowers or pods. It is premature to say that increased drought tolerance may contribute to larger seed or higher 100-seed weight. However, based on our result, the seed size of drought-stressed plants showed a strong association (R^2^ = 0.89) with the stress tolerance index. Upon rehydration, fewer seeds receiving abundant resources increased the seed size. A similar finding was reported by Ney et al^58^ in peas where drought stress reduced the number of seeds, but the retained seeds were larger.

Weaker relationship between yield components between treatments (control vs. drought, Table 1), suggests that cultivars with higher seed yields under control might not have a similar yield under drought^59^. Our data illustrated that a cultivar with an ability to withstand drought stress by modifying functional traits at flowering-seed setting might also confer the ability to recover from drought by retaining high pod number and pod weight. Based on these traits, cultivar G4620RX and DM45X61 exhibited better performance under drought stress (Fig. 7). These cultivars could be used for rainfed production or as donor parents to improve drought tolerance in soybean. A rapid accumulation of protein and oil occurs in soybean seeds during the pod-filling stage, which is often impacted by genotype and environment interaction^60–63^. We observed an increase in seed protein (4%) and a decrease in oil content (2%) under drought stress, similar to other study in soybean^31^. Irrespective of the genetic background, stressed plants attributed to the deposition of a substantial quantity of protein at the expense of the oil under drought stress^31^. This result indicates that improving protein and oil content simultaneously is challenging under stress^64,65^. Furthermore, different cultivars had different yield and quality characteristics, making it challenging to select one cultivar with high yield and quality. These results suggest a need to choose a network of structural and functional traits to improve stress tolerance in crops^66^.

## Conclusion

Our study showed considerable variation in physiology, yield and quality among soybean cultivars. We demonstrated that most of the tested soybean cultivars were sensitive to drought during flowering-early seed setting. Therefore, targeting a lower decrease in trait value or higher yield (seed number and weight) under drought would help select stable and superior cultivars. Identified drought-tolerant cultivars (G4620RX, LS5009XS, and R15-2422) confer the regulation of a combination of physiological, yield and quality traits under stress. These findings suggest that developing soybean cultivars for rainfed environments requires a combination of stress-adaptive traits. Variability in leaf reflectance properties and their relationship with physiological or yield components suggested that leaf-level sensing information can be used for phenotyping germplasm for stress tolerance.

## Materials and methods

### Plant materials and growth conditions

 Eight commercially available soybean cultivars differing in their yield potential along with two advanced breeding lines (R15-2422 and R01-416F, with improved yield and nitrogen fixation) were used in the study (Supplementary Table S1). The experiment was carried out from May to October 2021 at the Rodney Foil Plant Science Research Center of Mississippi State University, Mississippi, USA (33º28’ N, 88º47’ W) following the appropriate institutional guidelines. Four seeds were sown in 13.5 L pots (top diameter = 30 cm, bottom diameter = 23 cm, and height = 26 cm) filled with farm soil and grown under natural solar radiation. The seedlings were thinned to one plant per pot at the three-leaf stage. All the pots were fertilized with a controlled-release Osmocote (5 g) fertilizer (14–14-14 of N-P-K; Hummert International) before sowing and top-dressed (4 g) at the flowering stage. A systemic insecticide Marathon 1% G (Imidacloprid, OHP, Mainland, PA) was applied to each pot (4 g) after seedling emergence to avoid infestation of sucking pests. Plants were sprayed with Sanmite insecticide (Gowan Company, Yuma, Arizona, USA) at a rate of 0.5 g L^−1^ and Avid (Merck & Co, New Jersey, USA) at a rate of 3.32 g L^−1^ water to control mites inside the greenhouse during the experiment. All soybean plants were grown at a soil moisture content ~ 0.15 m^3^ m^−3^ volumetric water content (VWC) or irrigated optimally (100% evapotranspiration) for 50 days through an automated time-based pre-programmed drip irrigation.

### Stress treatments

Fifty days after sowing, pots (10 cultivars × 2 treatments × 10 replication = 200 pots) were transferred to greenhouse for stress imposition. One batch of hundred pots were maintained under control (32 °C day temperature, 100% irrigation characterized as irrigated) conditions. The other 100 pots were kept under drought conditions (32 °C day temperature, 50% irrigation, drought stress) for 30 days from flowering to the early seed setting stage (R1–R6). The experimental setup followed a 10 × 2 split-plot pattern for each cultivar with two irrigation treatments and ten replications within treatment in a randomized complete block design. Replicated soil moisture probes (Model EM5b Soil Moisture, Decagon Devices, Inc., Pullman, Washington, USA) were randomly set in the pots to monitor soil moisture at 15 cm depth periodically. In non-stress treatment, the VWC was above 0.15 m^3^ m^−3^ (100% irrigation) during the experimental period. Under drought stress, the VWC decreased to approximately 45 percent in 4 days after the beginning of drought stress. After 30 days of exposure to drought stress, all pots were rehydrated to reach 100% irrigation status similar to control and maintained until physiological maturity to record yield and quality components. The microclimatic conditions (temperature and relative humidity) were monitored at 15 min intervals throughout the experiment using HOBO data loggers (Onset Computer Corporation, Bourne, MA 02,532, USA) placed above the crop canopy.

### Data collection

#### In vitro pollen germination

 A week after stress imposition, fresh flowers from five random plants in each cultivar were collected between 09:00 am to10:00 am, and air-dried for 2 h. Pollen grains from each flower were dusted onto the germination medium to allow a uniform distribution of pollen grains on the surface of the medium^67^. The pollen germination medium consisted of 15 g sucrose (C_12_H_22_O_11_), 0.03 g calcium nitrate [Ca (NO_3_)_2_4H_2_O], and 0.01 g boric acid (H_3_BO_3_) dissolved in 100 ml of deionized water^68^. The chamber slides were then covered and incubated at 30 ºC (Precision Instruments, New York, USA) for three hours. The pollen grains were observed using a compound microscope at 40X magnification (AmScope with MU035 camera, California, USA). Pollen grain was considered germinated when its tube length was equal to or greater than the grain diameter^69^. Pollen germination percentage (PG %) was calculated using the following formula

$${\text{Pollen germination}}\% = \frac{{\text{Germinated pollen grains}}}{{\text{Total number of pollen grains}}} \times 100 \%$$

### Leaf pigments and physiological parameters

Leaf pigments (chlorophyll content, flavonoid index, anthocyanin index, and nitrogen balance index) were non-destructively measured using a handheld Dualex® Scientific instrument (Force A DX16641, Paris, France). The stomatal conductance (gsw), transpiration (E), and quantum efficiencies of photosynthetic electron transport through photosystem II (PhiPS2) were measured using a portable handheld LI-600 porometer system integrated with a fluorometer (LI-COR Biosciences, Lincoln, USA) across treatment between 10:00 am to 12:00 pm on sunny days. All measurements were taken on the ﻿young and third fully expanded trifoliate leaf 14 days after treatment. The canopy temperature was measured using infrared radiometers (Apogee Instruments Inc., Logan, UT 84,321, USA).

### Specific leaf area

After 15 days of stress imposition, the third fully expanded trifoliate leaf was collected randomly from five plants of each cultivar across treatments. The leaf area was measured using the LI- 3100 (LI-COR Biosciences, Lincoln, USA) area meter. The leaf dry weight was measured after drying at 75 °C for three days. The specific leaf area was calculated using the following equation

$${\text{Specific leaf area}} = \frac{Leaf surface area (cm^2)}{{\text{Leaf dry weight (g)}}} $$

### Spectral measurement

The leaf adaxial surface reflectance (350 to 2500 nm) data was collected on the third fully expanded trifoliate leaf at 14 days after stress using a PSR + 3500 spectroradiometer (Spectral Evolution, Massachusetts, USA) connected to a leaf clip assembly, which has an internal light source, with optical fiber cable. The white reference panel integrated with a leaf clip was used to calibrate the instrument before the start of the measurement and every half hour during the period of data collection. Each spectral reading comprised an average of ten complete scanning. The data were collected within ± 2 h of solar noon. Five sets of spectral bands (blue band–centered at 475 nm with a bandwidth of 32, the green band–centered at 560 nm with a bandwidth of 28, the red band–centered at 668 nm with a bandwidth of 16, the red-edge band–centered at 717 nm with a bandwidth of 12, and near-infrared band centered at 842 nm with a bandwidth of 58) similar to the commercially available Micasense RedEdge (MicaSense Inc., WA, USA) were binned to extract vegetation indices (VIs), similar to our previous study^44^. This spectral information was used to compute the VIs related to pigments and photosynthetic efficiency﻿: normalized difference red edge (NDRE)^70^, photochemical reflectance index (PRI)^71^, transformed chlorophyll absorption in reflectance index (TCARI)^72^ and visible atmospherically resistant index (VARI)^48^. The list of the vegetation indices along with their mathematical expression used in this study is listed in Supplementary Table S2.

### Yield components

Plant height was measured using a meter scale at the R8 stage when 95% of the pods reached mature pod color^73^. The number of branches and pods per plant were counted. The replicated plants were manually harvested to obtain the yield and yield components. The shoot and pods were separated from each plant. The pods were oven-dried at 35 °C for 24 h to maintain the uniform seed moisture content of 14% and threshed manually to obtain the seed weight. The number of seeds per plant was measured using a seed counter (NP5056-Model 850–2, LI-COR, Lincoln, NE, USA).

### Seed quality

The seed quality of soybean is determined using a Perten DA7250 (Perten Instruments, Springfield, IL, USA). The seed sample was poured into the stationary cup provided by Perten Instruments. The strike-off supplied by the company was used to remove the excess sample to make sure the sample did not exceed the upper edge of the dish. Each sample was scanned two times in reflectance mode using a stationary cup. The samples were mixed thoroughly before each scan, ensuring that the sample was representative of the given cultivar. The scanning was done using the default setting and calibrations developed by the DA 7250 manufacturer for soybean seed samples. From the manufacturer calibration manual for oilseeds, the coefficient of determination was 0.97 for protein, and 0.92 for oil^74^.

### Statistical analysis

Analysis of variance (ANOVA) was performed for all parameters to estimate the significance of treatment, cultivar, and their interaction using the library “agricolae” in RStudio 4.2.2 (https://www.R-project.org/, R Core). The experimental design was split plot randomized complete block design, with treatment as the main plot factor, and the cultivars as the subplot factor. Means were separated using LSD at *p* ≤ 0.05. The Pearson's correlation analysis was performed using the library “corrplot” and the balloon plot was generated using the library “ggpubr”. The stress tolerance index (STI) was calculated for all ten soybean cultivars using the formula defined by Fernandez^75^ .

$${\text{Stress tolerance index}} = \frac{Ys \times Yc}{{\left( {{\text{}} \,Xc \;{\text{}}} \right)^{2} }}$$

where Ys is the phenotypic mean of a given cultivar under drought stress; Yc is the phenotypic mean of a given cultivar under control and Xc = mean yield of all ten cultivars under control. A score from 1 (sensitive) to 10 (tolerant) was assigned to each cultivar based on the physiology, seed yield, and quality stress tolerance index. All graphs were generated using Sigma Plot 14.5 (Systat Software, San Jose, CA).

## Supplementary Information

Supplementary Information 1.

## Data Availability

The datasets used and/or analyzed during the current study are available from the corresponding author on reasonable request.
