# Supplementary material for: Resilience of soybean cultivars to drought stress during flowering and early-seed setting stages
Source: Sci Rep. 2023 Jan 23;13:1277. doi: 10.1038/s41598-023-28354-0 (PMC9870866; doi:10.1038/s41598-023-28354-0)
Supplement: Supplementary file 1 — Supplementary Information 1. [file 41598_2023_28354_MOESM1_ESM.docx]

**Resilience of soybean cultivars to drought stress during flowering and early-seed setting stages**

Sadikshya Poudel^1^, Ranadheer Reddy Vennam^1^, Amrit Shrestha^2^, K. Raja Reddy^1^, Nuwan K.

Wijewardane^2^, Krishna N. Reddy^3^, Raju Bheemanahalli^1^*

**Supplementary Table S1** Summary of soybean varieties used in the study.

| **Cultivar ​** | **Brand ​** | **MG** | **Remark** |
| --- | --- | --- | --- |
| 44-D49 ​ | Armor ​ | 4 ​ | Roundup Ready 2 Xtend |
| R15-2422 | University of Arkansas ​ | 4 ​ | Advanced breeding line |
| P46A86X ​ | Pioneer ​ | 4 ​ | Roundup Ready 2 Xtend |
| G4620RX ​ | AgriGold ​ | 4 ​ | Roundup Ready 2 Xtend |
| LS5009XS ​ | Local Seed | 5 ​ | Roundup Ready 2 Xtend |
| S48XT90 ​ | Dyna-Gro ​ | 4 ​ | Roundup Ready 2 Xtend |
| 4775E3S ​ | Progeny Ag ​ | 4 ​ | Enlist trait |
| DM 45X61 ​ | Donmario ​ | 4 ​ | Roundup Ready 2 Xtend |
| DG4825RR2/STS ​ | Delta grow ​ | 4 ​ | Late Roundup Ready |
| R01-416F ​ | ​ High protein | 4 ​ | Improved Yield and Nitrogen Fixation under drought Stress |

**Supplementary Table 2.** List of the vegetation indices and their mathematical expression used in this study.

| Vegetation indices | Equation | Reference |
| --- | --- | --- |
| Normalized Difference Rededge (NDRE) | $\frac{R_{840}-R_{717}}{N_{840}+R_{717}}$ | 70 |
| Photochemical Reflectance Index (PRI) | $\frac{R_{531}-R_{570}}{R_{531}+R_{570}}$ | 71 |
| Transformed Chlorophyll  Absorption In Reflectance Index (TCARI) | $3\times[\left( R_{700}-R_{670} \right)-0.2\times(R_{700}-R_{550})\times\frac{R_{700}}{R_{670}}$ | 72 |
| Visible Atmospherically  Resistant Index (VARI) | $\frac{R_{550}-R_{660}}{R_{550}+R_{660}-R_{470}}$ | 48 |
